# Transition of Bacterial Diversity and Composition in Tongue Microbiota during the First Two Years of Life

**DOI:** 10.1128/mSphere.00187-19

**Published:** 2019-05-29

**Authors:** Shinya Kageyama, Mikari Asakawa, Toru Takeshita, Yukari Ihara, Shunsuke Kanno, Toshiro Hara, Ichiro Takahashi, Yoshihisa Yamashita

**Affiliations:** a Section of Preventive and Public Health Dentistry, Division of Oral Health, Growth and Development, Faculty of Dental Science, Kyushu University, Fukuoka, Japan; b OBT Research Center, Faculty of Dental Science, Kyushu University, Fukuoka, Japan; c Section of Orthodontics and Dentofacial Orthopedics, Division of Oral Health, Growth and Development, Faculty of Dental Science, Kyushu University, Fukuoka, Japan; d Department of Pediatrics, Graduate School of Medical Sciences, Kyushu University, Fukuoka, Japan; University of Wisconsin—Madison

**Keywords:** development, infancy, oral microbiota, tongue

## Abstract

Newborns are constantly exposed to various microbes from birth; hence, diverse commensal bacteria colonize the oral cavity. However, how or when these bacteria construct a complex and stable ecosystem remains unclear. This prospective cohort study examined the temporal changes in bacterial diversity and composition in tongue microbiota during infancy. We longitudinally collected a total of 464 tongue swab samples from 8 infants (age of <6 months at baseline) for approximately 2 years. We also collected samples from 32 children (aged 0 to 2 years) and 73 adults (aged 20 to 29 years) cross-sectionally as control groups. Bacterial diversities and compositions were determined by 16S rRNA gene sequencing. The tongue bacterial diversity in infancy, measured as the number of observed operational taxonomic units (OTUs), rapidly increased and nearly reached the same level as that in adults by around 80 weeks. The overall tongue bacterial composition in the transitional phase, 80 to 120 weeks, was more similar to that of adults than to that of the early exponential phase (EEP), 10 to 29 weeks, according to analysis of similarities. Dominant OTUs in the EEP corresponding to Streptococcus peroris and Streptococcus lactarius exponentially decreased immediately after EEP, around 30 to 49 weeks, whereas several OTUs corresponding to Granulicatella adiacens, Actinomyces odontolyticus, and Fusobacterium periodonticum reciprocally increased during the same period. These results suggest that a drastic compositional shift of tongue microbiota occurs before the age of 1 year, and then bacterial diversity and overall bacterial composition reach levels comparable to those in adults by the age of 2 years.

**IMPORTANCE** Evaluating the development of oral microbiota during infancy is important for understanding the subsequent colonization of bacterial species and the process of formation of mature microbiota in the oral cavity. We examined tongue microbiota longitudinally collected from 8 infants and found that drastic compositional shifts in tongue microbiota occur before the age of 1 year, and then bacterial diversity and overall bacterial composition reach levels comparable to those in adults by the age of 2 years. These results may be helpful for preventing the development of various diseases associated with oral microbiota throughout life.

## Abstract (precis)

Evaluating the development of oral microbiota during infancy is important for understanding the subsequent colonization of bacterial species and the process of formation of mature microbiota in the oral cavity. We examined tongue microbiota longitudinally collected from 8 infants and found that drastic compositional shifts in tongue microbiota occur before the age of 1 year, and then bacterial diversity and overall bacterial composition reach levels comparable to those in adults by the age of 2 years. These results may be helpful for preventing the development of various diseases associated with oral microbiota throughout life.

## INTRODUCTION

The oral cavity is one of the largest bacterial reservoirs in the human body, and numerous coexisting bacteria construct a complex and stable bacterial community (1–4). It is well known that mutans streptococci and the red complex, which includes Porphyromonas gingivalis, Tannerella forsythia, and Treponema denticola, are the most important bacteria associated with dental caries and periodontal disease, which are major oral diseases (5–9). Additionally, the existence of specific oral bacterial species related to systemic diseases such as obesity, cardiovascular diseases, and pneumonia have been reported in recent years (10–16). In contrast, oral dysbiosis (imbalance of overall bacterial composition including oral commensal bacteria and pathogenic bacteria) has attracted attention as an etiology of oral and systemic diseases (3, 4, 17–19).

After birth, the oral cavity of newborns is exposed to a wide variety of microbes, and the oral microbiota develops as newborns grow. This development begins with tolerated colonization of specific microbes in the human oral cavity. It is thought that facultative anaerobic bacteria such as *Streptococcus* and *Actinomyces* are pioneer colonizers in a newborn’s oral cavity; particularly, Streptococcus salivarius is thought to be the most predominant pioneer colonizer (20). Although the phylum *Firmicutes* predominates in both oral and gut microbiota, the genus *Streptococcus* within the phylum *Firmicutes* is rarely detected in gut microbiota (1). Thus, the predominance of *Streptococcus* species is characteristic of the first step in forming oral microbiota. It has also been reported that formation of the oral indigenous microbiota begins within the first 6 weeks of life, and *Streptococcus* rapidly dominates the oral cavity during this stage (21).

A recent cross-sectional study investigated subjects with different dentition states, including the predentate stage (age, 0 years) and primary dentition stage (age, 0 to 5 years). The results revealed a drastic expansion of bacterial diversity after tooth eruption (22). The authors also suggested that certain bacterial species were acquired in the predentate stage as consistent core species of human oral microbiota. Given that these early bacterial communities, including core species and species that immediately colonize after primary tooth eruption, lead to subsequent colonization as a foundation and affect the formation of adult oral microbiota related to various diseases in later life, it is important to understand bacterial acquisition during this period. However, the detailed characteristics of the acquisition process and temporal shift of oral microbiota composition and diversity during this period remain unclear.

In this prospective cohort study, we focused on the development of tongue microbiota during infancy. We analyzed tongue microbiota collected from 8 infants at short intervals by 16S rRNA gene amplicon deep sequencing and followed the infants’ transitions from 6 months to approximately 2 years of age. The aim of this study was to clarify temporal changes in infant tongue microbiota in detail and to reveal the characteristics of tongue microbiota in infancy compared to those in adults.

## RESULTS

### Study subjects and sequence.

Tongue swab samples were collected longitudinally from 8 infants, aged 13 to 26 weeks at baseline, for 82 to 98 weeks. A total of 464 samples (average, 58.0 samples per subject; range, 33 to 88 samples) were obtained. The characteristics and details of follow-up in each subject are presented in Table 1. We also collected cross-sectional tongue swab samples from 32 children and 73 adults. We analyzed these samples by 16S rRNA gene amplicon analysis, resulting in 10,175,374 high-quality reads (17,883 ± 11,918 reads per sample) to determine their bacterial compositions and diversities. The data were further analyzed to characterize the development of tongue microbiota in infants.

**TABLE 1:** Characteristics of the subjects

Subject no.^*a*^	Sex^*b*^	Wt at birth (g)	Type of feeding^*c*^	Use of antibiotics (mo)	Eruption of teeth (mo)	Start of baby food (mo)	Start of outside play (mo)	Sampling period (wk)	No. of samples
1	F	2,896	Breast		9	6	11	13–111	45
2	M	2,952	Breast		5	5	14	15–109	62
3	F	2,987	Mix (breast)		7	7	13	17–111	88
4	F	1,885	Mix (artificial)		7	7	12	20–108	45
5	F	1,320	Mix (artificial)	3	7	7	12	20–108	43
6	M	3,046	Breast	10, 16, 18	6	6	14	21–113	69
7	F	2,734	Breast	2	5	5	11	22–112	79
8	F	3,028	Breast		6	6	10	26–108	33

a Subjects 4 and 5 were identical twins.

b F, female; M, male.

c The main type of feeding is given in parentheses.

### Transition of bacterial diversity in tongue microbiota.

**FIG 1:**
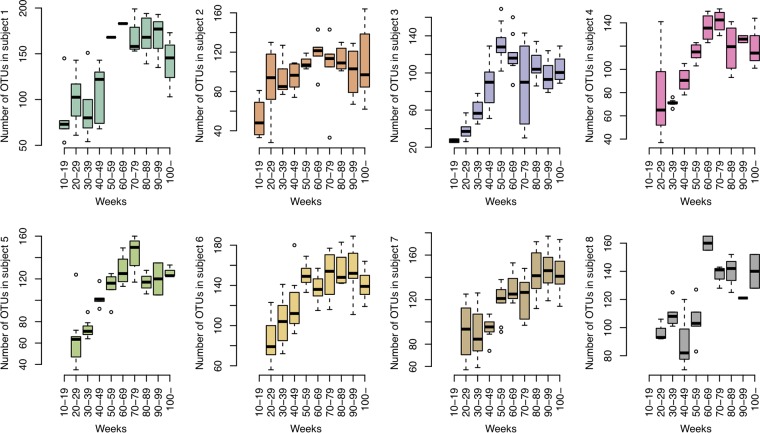
Transition of bacterial diversity in each subject. Box plots show the number of OTUs in each period.

**FIG 2:**
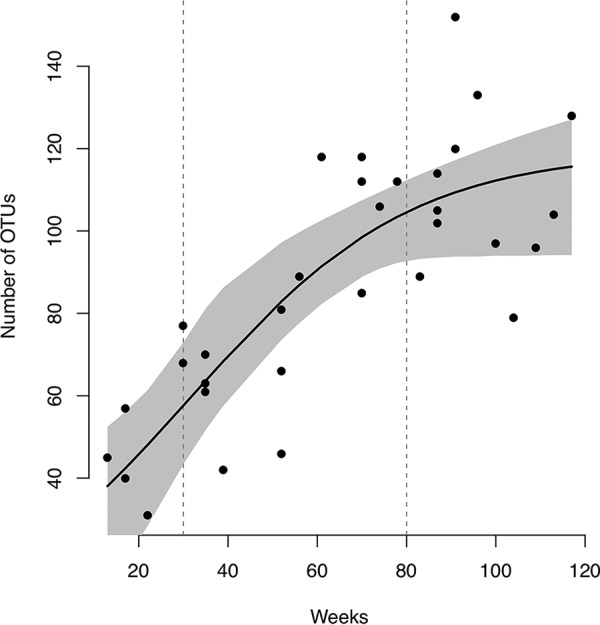
Transition of bacterial diversity in the cross-sectional control group. Plots show the number of OTUs in each child. The black curve indicates a logistic curve fitted using the NLS function based on the self-start logistic (SSlogis) model. The gray area indicates the 95% confidence interval of the logistic curve.

We first investigated the number of observed operational taxonomic units (OTUs) in tongue microbiota to evaluate the maturity of tongue bacterial populations. The number of OTUs detected from tongue microbiota rapidly increased with age until 80 weeks (Fig. 1). In the cross-sectional samples from children, a similar increasing tendency in the number of OTUs was observed until approximately 80 weeks of age (Fig. 2). We defined 10 to 29 weeks as the early exponential phase (EEP), corresponding to the initial stage of increase, and 80 to 120 weeks as the transitional phase (TP), corresponding to the phase between exponential and stationary stages in infancy. When bacterial alpha diversity was compared between the EEP and TP, the number of OTUs in the TP was significantly higher than that in the EEP in all subjects (Mann-Whitney U test) (Table 2). Interestingly, the number of OTUs in the TP was either not significantly different or significantly higher than that of adult samples in all subjects except for subject 3. This result suggests that the bacterial alpha diversity of tongue coating in the TP had reached the same level as that in the adult phase.

**TABLE 2:** Number of OTUs in the EEP and TP in each subject^*a*^

Subject no. or group	Mean no. of OTUs (SD)		*P* value^*a*^	No. of OTUs in TP vs adult phase (*P* value)^*b*^
	EEP	TP		
1	93.88 ± 28.20	159.83 ± 27.57	<0.001	Significantly high (<0.001)
2	79.64 ± 33.67	107.74 ± 26.75	0.028	NS (0.261)
3	36.50 ± 9.66	102.87 ± 14.63	<0.001	Significantly low (0.003)
4	74.50 ± 33.11	120.44 ± 17.51	0.009	NS (0.436)
5	64.0 ± 24.08	121.25 ± 11.30	0.002	NS (0.289)
6	87.11 ± 22.11	148.09 ± 20.41	<0.001	Significantly high (<0.001)
7	91.88 ± 25.11	144.94 ± 17.81	<0.001	Significantly high (<0.001)
8	97.0 ± 7.81	136.67 ± 13.82	0.028	Significantly high (0.009)
Controls^*c*^	43.25 ± 10.84	109.92 ± 20.50	0.001	NS (0.370)

a Significant differences were determined using a Mann-Whitney U test.

b The number of OTUs in adult samples is 115.12 ± 20.16. NS, not significant (Mann-Whitney U test).

c Cross-sectional samples.

### Similarities of bacterial compositions between phases.

**FIG 3:**
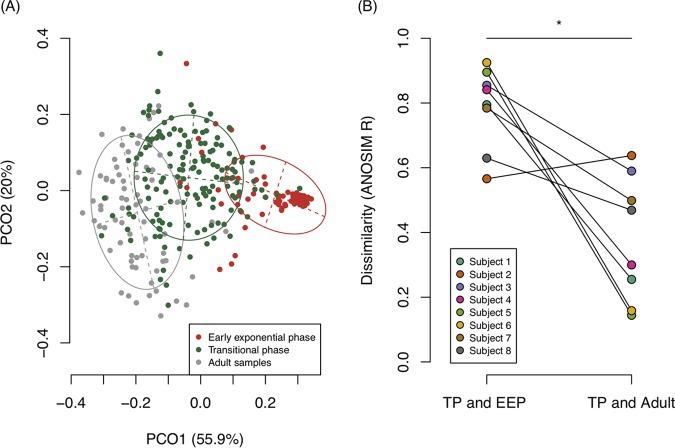
(A) Principal-coordinate analysis (PCoA) plot of tongue bacterial composition belonging to each phase in all subjects. The samples belonging to each community type are identified according to the color legend. These two components explained 75.9% of variance. The intersection of the broken lines indicates the center of gravity for each community type. Each ellipse covers 67% of the samples belonging to each community type. (B) Dissimilarities between phases in each subject. Dissimilarity was evaluated by the analysis of similarities (ANOSIM) *R* (range from −1 to 1), with values close to 1.0 indicating dissimilarity between groups and a value of 0 indicating completely random sampling. Plots on the left indicate the ANOSIM *R* between the early exponential phase (EEP; 0 to 29 weeks) and translational phase (TP; 80 to 120 weeks), and plots on the right indicate the ANOSIM *R* between the TP and young-adult controls. Subjects are represented according to the color legend. Significant differences were determined using Wilcoxon’s signed-rank test. *, *P* < 0.05.

Figure 3A shows a principal-coordinate analysis (PCoA) plot of the tongue bacterial compositions of all samples belonging to the EEP, TP, and adult phase. Plots of the EEP clearly differed from those of the TP and adult samples. Furthermore, we statistically estimated the similarities (dissimilarities) of bacterial compositions between the EEP, TP, and adult samples using analysis of similarities (ANOSIM) based on the weighted UniFrac distance. ANOSIM can statistically reveal significant differences in bacterial compositions between groups, and an *R* value (ranging from −1.0 to 1.0) close to 1.0 suggests maximum dissimilarity. ANOSIM showed that the overall bacterial composition of the TP was more similar to that of the adult samples (analysis of similarity *R* = 0.326) than that of the EEP (*R* = 0.489). Moreover, when samples were compared individually as shown in Fig. 3B, the similarities between the TP and adult samples were significantly higher (*R* value range, 0.14 to 0.64) than those between the TP and EEP samples (*R* value range, 0.57 to 0.92) (Wilcoxon’s signed-rank test, *P* = 0.016) although the former comparison used data from a single individual while the latter used data of different individuals. These results indicate that the bacterial composition drastically changed between the EEP and TP and that the bacterial composition of the TP was already more similar to that of the adult phase than to that of the EEP.

### Dominant OTUs and transition of their abundances.

**FIG 4:**
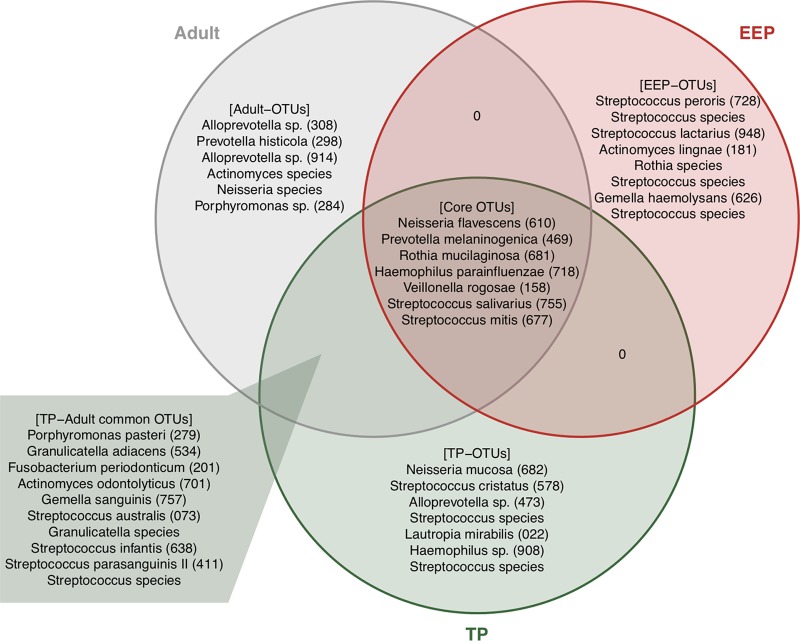
Distribution of dominant OTUs among each phase using a Venn diagram. Dominant OTUs showing ≥1% of the mean relative abundance in the early exponential phase (EEP), transitional phase (TP), and adult phase are shown. EEP-, TP-, and adult-OTUs are OTUs dominant only in the respective individual phase. TP-adult common OTUs show dominance in both the TP and adult phase. Core OTUs were dominant in all phases. Oral taxon identification numbers from the HOMD are shown in parentheses following bacterial names.

We examined the temporal changes in bacterial composition in tongue microbiota at the species level to clarify the details of bacterial colonization and substitution during the early developmental stage. First, we identified dominant OTUs with a ≥1% mean relative abundance in the EEP, TP, and adult phase, and summarized their distributions among these phases using a Venn diagram (Fig. 4). Seven OTUs corresponding to bacterial species such as Neisseria flavescens HOT-610 (human oral taxon numbers in Human Oral Microbiome Database [HOMD]), Prevotella melaninogenica HOT-469, and Rothia mucilaginosa HOT-681 were commonly dominant in all phases (core OTUs). In addition, 10 OTUs corresponding to bacterial species such as Porphyromonas pasteri HOT-279, Granulicatella adiacens HOT-534, and Fusobacterium periodonticum HOT-201 were commonly dominant in both the TP and adult phase (TP-adult common OTUs). No common OTUs were observed in the EEP and TP except for core OTUs. This finding is consistent with the above result of ANOSIM showing that the bacterial composition in the TP was more similar to that in the adult phase than to that in the EEP.

**FIG 5:**
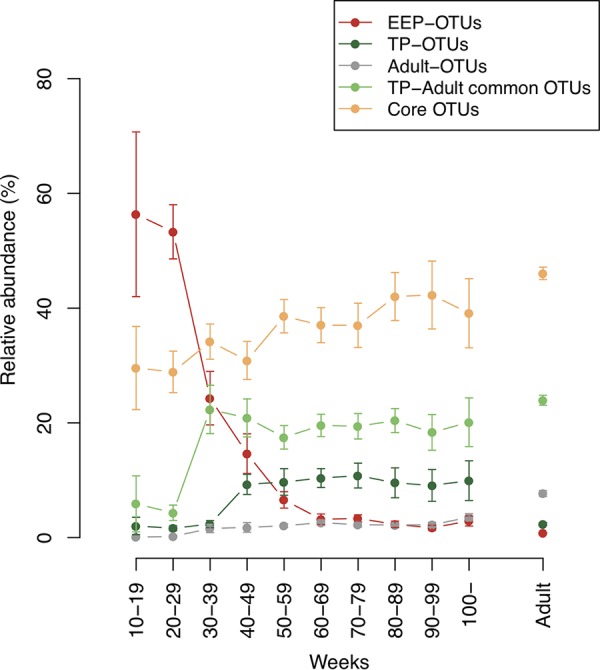
Transition of bacterial composition of dominant OTUs. EEP-, TP-, and adult-OTUs indicate OTUs dominant only in the respective individual phase. TP-adult common OTUs have dominance in both the TP and adult phase. Core OTUs were dominant in all phases. Each line plot indicates a shift in the total relative abundance. Each plot shows the average of the total relative abundance of each group of dominant OTUs in each period, and error bars show standard errors.

Thereafter, we studied the temporal shift in the total relative abundance of dominant OTUs according to Venn diagram analysis. Core OTUs showed a high relative abundance in the range of 28.89 to 42.29% in all phases (Fig. 5). Eight OTUs that were dominant only in the EEP (EEP-OTUs) consisted of OTUs corresponding to bacterial species such as Streptococcus peroris HOT-728 and Streptococcus lactarius HOT-948, and their total relative abundance exponentially decreased immediately after the EEP, particularly around 30 to 49 weeks, from approximately 60% to below 3%. Additionally, TP-adult common OTUs rapidly increased during this phase, accounting for approximately 20% of tongue microbiota after that, even in the adult phase. These results suggest that a drastic change occurs in the bacterial composition before the age of 1 year.

## DISCUSSION

This prospective cohort study revealed the early development of bacterial diversity and composition of tongue microbiota. The drastic compositional shift in tongue microbiota occurred in an early stage of infancy, around 30 to 49 weeks, and then the bacterial diversity and overall bacterial composition in the TP reached the same levels as those in adults and were less comparable to those in the EEP. The result is particularly interesting considering that the TP was only approximately 1 year later than the EEP and that the comparison between the TP and EEP was carried out within the same individual although the comparison between the TP and the adult phase was conducted among different individuals.

A recent cross-sectional study suggested that oral bacterial diversity in the primary dentition period is significantly higher than that in the predentate stage (22). Although we did not consider the effect of primary dentition because of a lack of sufficient edentulous samples, this prospective cohort study revealed that expansion of the bacterial diversity in the oral cavity may begin during the earliest phase of the primary dentition period. These results suggest that tongue microbiota drastically develop and approach maturity immediately after eruption of the primary teeth. Additionally, dominant OTUs in adults, such as N. flavescens, P. melaninogenica, and R. mucilaginosa, were already detected and colonized in an early stage of infancy as core bacterial species. These results suggest that the foundation of tongue microbiota is established in infancy.

Although we frequently collected samples at short intervals and obtained information from several events to identify factors affecting the development of tongue microbiota, an obvious factor affecting the drastic increase of bacterial alpha diversity and compositional changes in bacterial species was difficult to identify because of the small number of subjects. Additionally, samples from patients that would contribute to understanding the origins of oral bacteria in infants were not collected. Further studies involving larger sample sizes focusing on early infancy are required to identify the factors that critically affect the development of oral microbiota.

A set of twins (subjects 4 and 5) among our subjects showed interesting results. Their parents began the feeding of baby food starting at 7 months; while one (subject 4) child quickly accepted the baby food, the other (subject 5) did not accept this food until it was approximately 1 year old. However, there was no drastic difference in the development of bacterial diversity and compositional shift between the twins. These results suggest that the change in nutrition from lactation to weaning food did not greatly affect the composition of tongue microbiota and that environmental factors other than eruption of primary teeth or genetic factors may have stronger effects on temporal changes in tongue microbiota from the EEP to TP.

Our study revealed that the bacterial composition of the TP was more similar to that of the adult phase than to that of the EEP according to ANOSIM. ANOSIM also revealed a significant difference in bacterial compositions between the TP and adult phase in most subjects (data not shown). Indeed, seven OTUs corresponding to bacterial species such as Neisseria mucosa HOT-682 and Streptococcus cristatus HOT-578 were dominant in only the TP (TP-OTUs), whereas six OTUs corresponding to bacterial species such as *Alloprevotella* species strain HOT-308 and Prevotella histicola HOT-298 were inversely dominant in only adult samples (adult-OTUs). These OTUs were thought to be discriminative OTUs between the TP and adult phase. More interesting is that Streptococcus cristatus and Lautropia mirabilis HOT-022, which are predominant in supragingival plaque compared to their presence at other oral sites in the adult phase (23, 24), were dominant in the TP, suggesting that the emergence of tooth surfaces contributes to colonization of these microbes in the oral cavity. These microbes disappeared from the tongue coating of adults, even though there are fewer teeth in the TP than in adults. Although we could not confirm whether these microbes were present in other niches because we examined only tongue swabs in this study, the reduction in the populations of S. cristatus and L. mirabilis in the tongue coating may not mean that they are absent from the oral cavity; these microbes tightly colonize tooth surfaces by coaggregating with new inhabitants in the adult phase. Alternatively, unknown factors emerging during growth enhance the tight colonization of these microbes in supragingival plaque, which results in the reduction of the relative abundances of these microbes in the tongue coating and saliva. If the latter hypothesis is true, identification of such factors would reveal a new mechanism by which colonization of oral bacteria and formation of dental plaque occur.

*Streptococcus* is among the most dominant genera in the human oral cavity, and previous reports of oral microbiota of infants demonstrated that S. salivarius is frequently detected in the infant oral cavity (20, 22). In fact, S. salivarius belonged to the core OTUs and showed a high relative abundance staring at the EEP (Fig. 4 and Fig. S1 in the supplemental material). In contrast, S. peroris, S. lactarius, and other *Streptococcus* species belonging to the EEP-OTUs exponentially decreased and disappeared by the TP. This result suggests that substitution of bacterial species, even in the same genus, occurred in tongue microbiota during this period. Interestingly, an OTU that nearly corresponded to S. salivarius (97.2% identity against the HOMD) was dominant in the EEP of some subjects but disappeared from the oral cavity in the TP and adult phase. This suggests that there are two types of S. salivarius with slightly different base sequences and different behaviors residing in or leaving the oral cavity. Further studies of these bacterial species using comparative genomics may reveal the detailed colonization mechanisms of oral commensal bacteria.

10.1128/mSphere.00187-19.1 FIG S1 **Transition of bacterial composition of each dominant OTU.** EEP-, TP-, and Adult-OTUs indicate dominance in each phase only. TP-Adult common OTUs indicate dominance in both the TP and adult phase. Core OTUs were dominant in all phases. Each OTU is depicted using a different color. Download FIG S1, TIF file, 0.6 MB. Copyright © 2019 Kageyama et al. 2019 Kageyama et al. This content is distributed under the terms of the Creative Commons Attribution 4.0 International license.

While 16S rRNA gene sequencing may not be suitable for detecting specific bacteria present at low proportions, we examined abundances of Porphyromonas gingivalis and Streptococcus mutans, which are the major pathogens of periodontitis and dental caries, in each subject (Fig. S2). As a result, we detected P. gingivalis in the tongue coating of most subjects from a very early period, even when the subjects did not have periodontitis or teeth. Additionally, P. gingivalis disappeared by the TP in most subjects, but subjects 1 and 8 had evidence of P. gingivalis until the TP. These results suggest that the tongue dorsum harbors periodontal pathogens as a reservoir starting in early infancy. In contrast, the detection of S. mutans was transient and unstable compared to that of P. gingivalis.

10.1128/mSphere.00187-19.2 FIG S2 **Detection and abundance of oral pathogens in infancy.** The *y* axis shows each longitudinal subject, and the *x* axis shows each time point. Circle size means detection rates of pathogens among samples belonging to each time point in each subject, and circle color indicates their mean relative abundances. The detection rates were calculated following rarefaction to 4,000 reads per sample. Download FIG S2, TIF file, 0.3 MB. Copyright © 2019 Kageyama et al. 2019 Kageyama et al. This content is distributed under the terms of the Creative Commons Attribution 4.0 International license.

Although the number of subjects was small in the present study, no previous studies have evaluated an average of 58 tongue samples per individual, collected consistently for approximately 2 years, based on next-generation sequencing. To compensate for the small sample size of our study, we validated our results by utilizing cross-sectional control samples. Therefore, the results obtained in the present study appear to be generalized to the development of tongue microbiota in infancy in Japanese infants.

In conclusion, the present longitudinal study demonstrated that the bacterial diversity and composition of tongue microbiota rapidly develop in an early stage of infancy. To prevent the development of oral and systemic diseases associated with oral microbiota, oral care based on a bacteriological basis, e.g., changing the oral microbiota from a dysbiotic state to a healthy state or maintaining a healthy state, is indispensable in future preventive health care. Although little is known about how to change the oral microbiota, the monitoring and intervention of infant oral microbiota, particularly until the first year of life, may help to prevent the development of various diseases later in life.

## MATERIALS AND METHODS

### Study subjects.

We enrolled 8 infants (2 male and 6 female), aged 13 to 26 months, living in Fukuoka City, Fukuoka Prefecture, Japan, for a longitudinal study spanning approximately 2 years. To confirm the results of the longitudinal study, 32 healthy children (18 male and 14 female; age, 0 to 2 years old) who had not used antibiotics within a month preceding sampling were also enrolled as cross-sectional controls from our previous project of disease in children. As a reference adult control, 73 healthy participants (36 men and 37 women; age, 20 to 29 years), consisting of dental students at Kyusyu University who had not used antibiotics within a month preceding sampling, were also enrolled from our previous project of dental plaque. Written informed consent was obtained from all subjects or their parents. The ethics committee of Kyushu University approved this study and the informed consent procedure (approval numbers 22-146, 25-110, and 28-127).

### Sample of microbiota and data collection.

Tongue swab samples were collected from all children by scraping the tongue dorsum with a cotton swab. Longitudinal samples were collected by their parents and immediately stored in the home freezer. Samples were transported to our laboratory and stored at −30°C until further analysis. Feeding information (breast milk, artificial milk, or baby food), eruption of teeth, start of outside play, and antibiotic administration were recorded by the parents. Tongue swab samples from adults were collected using a circular bonded-fiber fabric with a diameter of 15 mm as described previously (4). These samples were immediately stored at −30°C until further analysis.

### 16S rRNA gene amplicon sequencing analysis of tongue swab samples.

Tongue swab samples were examined by 16S rRNA gene next-generation sequencing using an Ion PGM system (Thermo Fisher Scientific, Waltham, MA, USA). DNA was extracted from the samples collected from children using the bead-beating method (25), and DNA was extracted from samples collected from adults using lysozyme and achromopeptidase (26), as described previously. The V1-V2 regions of the 16S rRNA gene were amplified using the following primers: 8F (5′-AGA GTT TGA TYM TGG CTC AG-3′) with the Ion Torrent adaptor A and a sample-specific 8-base tag sequence and 338R (5′-TGC TGC CTC CCG TAG GAG T-3′) with the Ion Torrent trP1 adaptor sequence. PCR amplification, purification, and quantification of each PCR amplicon were performed as previously described (3). Equal amounts of the purified PCR amplicons were pooled, and gel purification was carried out using a Wizard SV Gel and PCR Clean-Up System (Promega, Madison, WI, USA). The DNA concentration was determined using a KAPA Library Quantification kit (KAPA Biosystems, Wilmington, MA, USA), and DNA was diluted to 8 pM for use as the template DNA for emulsion PCR. Emulsion PCR and enrichment of template-positive particles were performed using an Ion PGM Template OT2 400 kit and Hi-Q View OT2 kit (Thermo Fisher Scientific) in an Ion One Touch 2 system (Thermo Fisher Scientific). The enriched particle was loaded onto 10 Ion 318 v2 chips (Thermo Fisher Scientific), and sequencing was performed on the Ion PGM (Thermo Fisher Scientific) using an Ion PGM Hi-Q sequencing kit and Hi-Q view sequencing kit (Thermo Fisher Scientific).

### Data analysis and taxonomy assignment.

We conducted quality filtering of the raw sequence reads using a script written in R (version 3.5.1). The reads were excluded from the analysis when they were ≤200 bases, had an average quality score of ≤25, did not include the correct forward primer sequence, did not include the correct reverse primer sequence (one mismatch was allowed), or had a homopolymer run of >6 nucleotides (nt). The quality-checked reads (including singleton reads) were assigned to the appropriate sample by examining the tag sequence, and then forward and reverse primer sequences were trimmed. OTUs were constructed by clustering quality-checked reads, excluding singleton reads, with a minimum pairwise identity of 97% using UPARSE (27) as described previously (3). All quality-checked reads (including singleton reads) were mapped to each OTU with an identity of ≥97% using UPARSE (3). The taxonomy of representative sequences was determined using BLAST against 889 oral bacterial 16S rRNA gene sequences (HOMD 16S rRNA RefSeq, version 14.51) in the Human Oral Microbiome Database (28). Nearest-neighbor species with ≥98.5% identity were selected as candidates for each representative OTU. The taxonomy of sequences without hits was further determined using the Ribosomal Database Project (RDP) classifier with a minimum support threshold of 80%. The number of OTUs and UniFrac distance (29) were calculated following rarefaction to 4,000 reads per sample using R.

### Statistical analysis.

A logistic curve was fitted to the transition data of the bacterial diversities (number of OTUs) in tongue microbiota using the nonlinear least squares (NLS) function, based on the self-start logistic (SSlogis) model that can avoid the step of determining an initial value in the R package (30). The 95% confidence interval of the logistic curve was estimated using the predictNLS function in the propagate library of R. We compared the number of OTUs between different phases using a Mann-Whitney U test. The UniFrac metric was used to determine the dissimilarity between any pair of bacterial compositions. Principal-coordinate analysis (PCoA) was performed based on the weighted UniFrac distance. We estimated the *R* value between phases by analysis of similarities (ANOSIM) with 999 permutations based on the weighted UniFrac distance in each subject and compared the *R* values using Wilcoxon’s signed-rank test.

### Data availability.

The sequence data have been deposited in DDBJ/NCBI Sequence Read Archive under accession number DRA008297.
