# Supplementary figures and images for: Transition of Bacterial Diversity and Composition in Tongue Microbiota during the First Two Years of Life
Source: mSphere. 2019 May 29;4(3):e00187-19. doi: 10.1128/mSphere.00187-19 (PMC6541735; doi:10.1128/mSphere.00187-19)

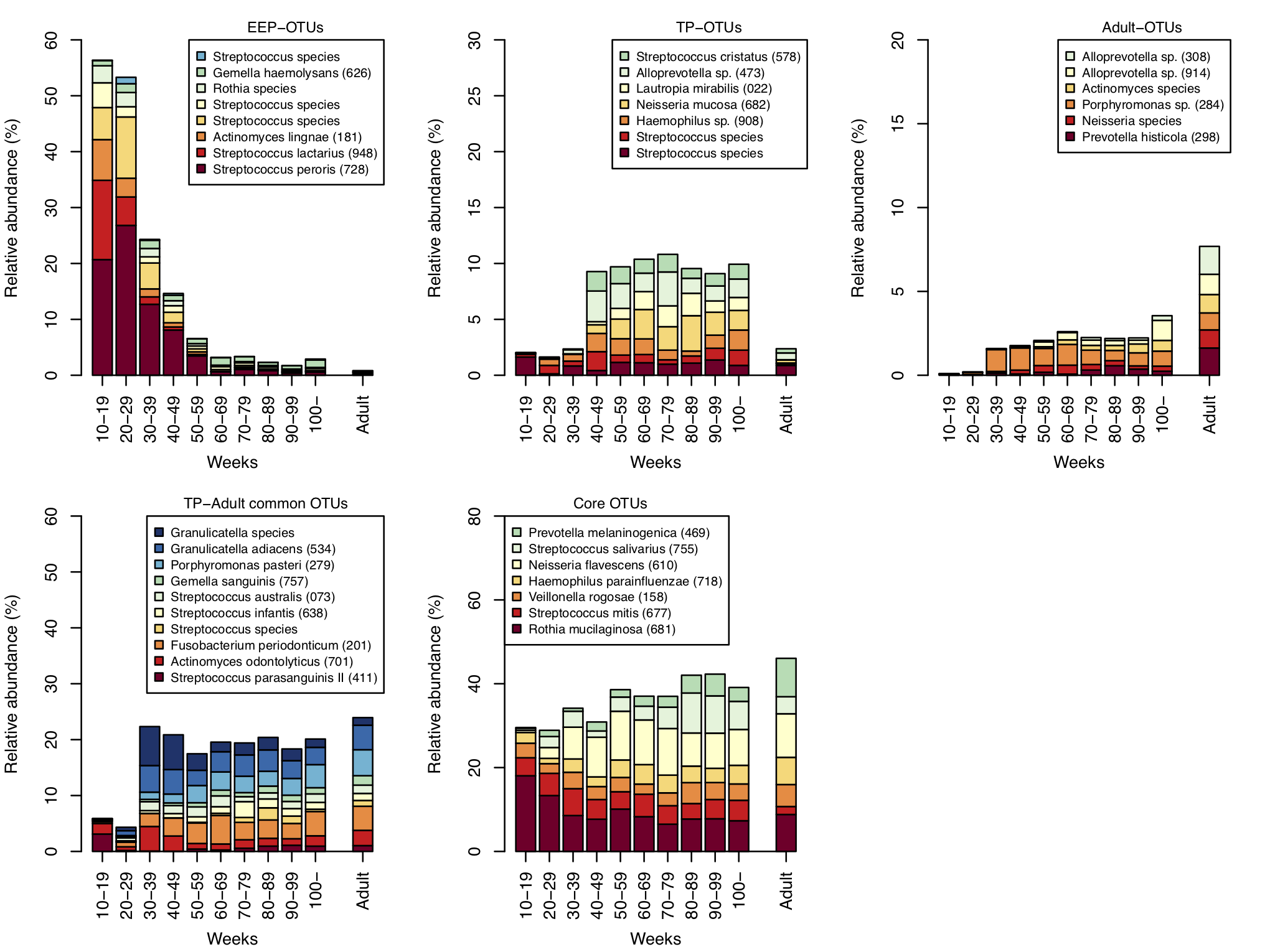

Supplement: FIG S1 [file mSphere.00187-19-sf001.tif]

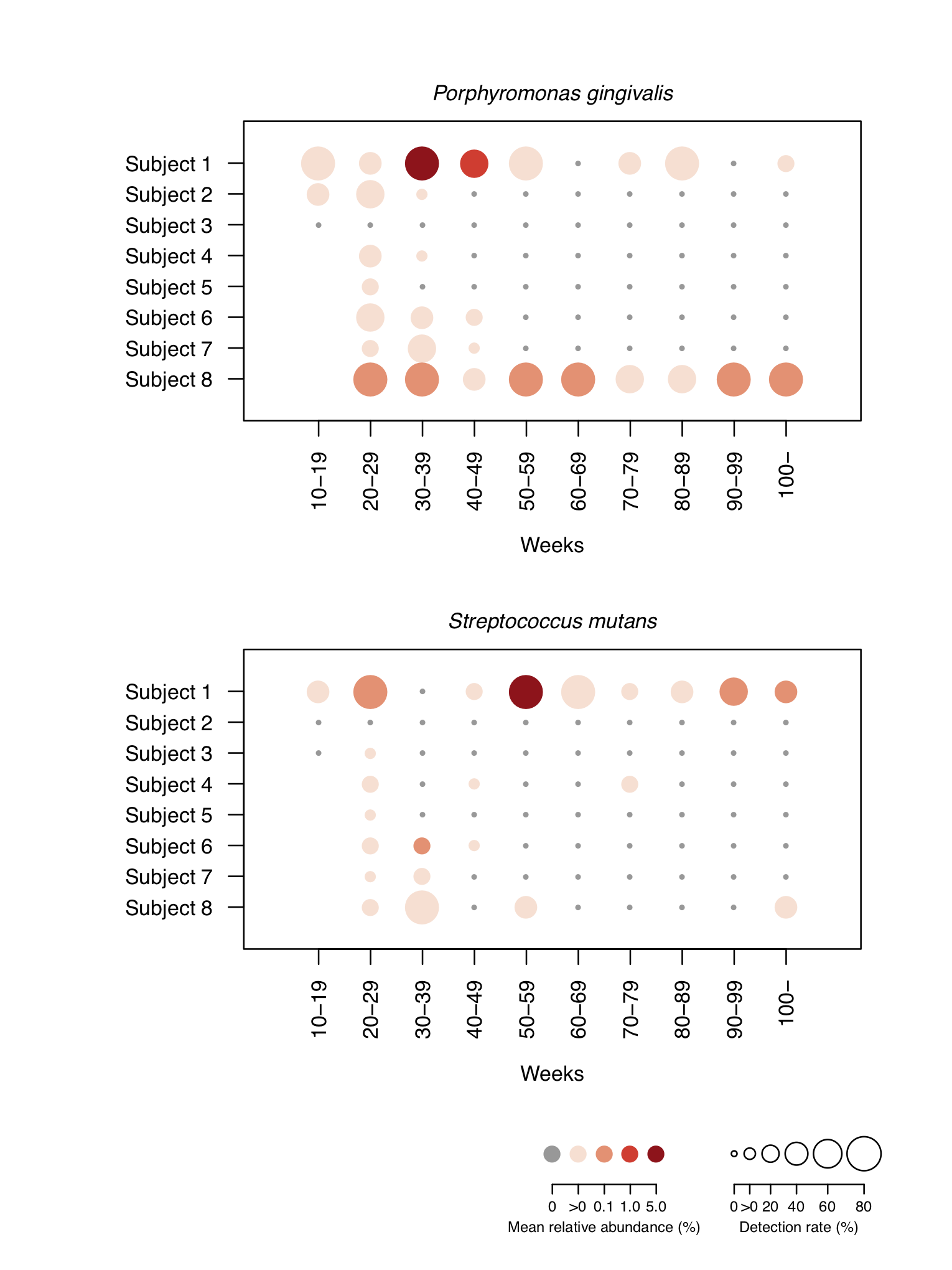

Supplement: FIG S2 [file mSphere.00187-19-sf002.tif]
